# Real-time PCR Machine System Modeling and a Systematic Approach for the Robust Design of a Real-time PCR-on-a-Chip System

**DOI:** 10.3390/s100100697

**Published:** 2010-01-19

**Authors:** Da-Sheng Lee

**Affiliations:** Department of Energy and Refrigerating Air Conditioning Engineering, National Taipei University of Technology, Taipei, Taiwan; E-Mail: f11167@ntut.edu.tw; Tel.: +886-2-2771-2171 ext 3510; Fax: +886-2-2731-4919

**Keywords:** DNA quantification reliability, robust design, system identification model, real-time PCR machine, real-time PCR on-a-chip

## Abstract

Chip-based DNA quantification systems are widespread, and used in many point-of-care applications. However, instruments for such applications may not be maintained or calibrated regularly. Since machine reliability is a key issue for normal operation, this study presents a system model of the real-time Polymerase Chain Reaction (PCR) machine to analyze the instrument design through numerical experiments. Based on model analysis, a systematic approach was developed to lower the variation of DNA quantification and achieve a robust design for a real-time PCR-on-a-chip system. Accelerated lift testing was adopted to evaluate the reliability of the chip prototype. According to the life test plan, this proposed real-time PCR-on-a-chip system was simulated to work continuously for over three years with similar reproducibility in DNA quantification. This not only shows the robustness of the lab-on-a-chip system, but also verifies the effectiveness of our systematic method for achieving a robust design.

## 1. Introduction

Wittwer *et al*. first began to develop real-time Polymerase Chain Reaction (PCR) machines in 1997 [1,2]. This type of instrument has an integrated fluorimeter to allow in-tube real-time analysis of DNA template samples. A rise in fluorescence of the signal of each cycle indicates amplification. The ability to monitor reaction progress has a number of advantages over endpoint analysis. As a result, real-time PCR has proven to be a powerful tool for genetic analysis [3,4].

Micro-Electronic Mechanical System (MEMS) technologies are being developed in the semiconductor industry, and the characteristic dimensions of the small structures used are on the order of μm. The Samsung Advanced Institute of Technology Bio Lab reported a real-time PCR-on-a-chip system in 2006 [5]. They also demonstrated the successful screening of HBV-infected patients using this lab-on-a-chip system. Since then much research has been published on the development of real-time PCR on-a-chip systems [6–11].

Real-time PCR-on-a-chip systems for DNA quantification are expected to be a common home health-care tool in the future. In point-of-care testing, however, this type of instrument may not be maintained or calibrated regularly. Since machine reliability is the key issue for normal operation, this study develops a systematic approach to improving the reliability of the lab-on-a-chip system.

## 2. Methods

### 2.1. Robust Design

The robust design method was first introduced by Dr. Genichi Taguchi [12]. Many reliable products were created using this method in various industries: automobiles, telecommunications, electronics, software, etc. In this study, robust design was applied to the real-time PCR on-a-chip. By creating a design less sensitive to various causes of variation, this methodology provides a solution for producing a reliable instrument design.

### 2.2. Systematic Approach for the Robust Design of a Real-Time PCR-on-a-Chip System

In developing a real-time PCR-on-a-chip system, the following mathematical tools for robust design were adopted.

- The system identification model [13]: A system identification model was built as a guide for ideal function. This model specifies the signal factor of the real-time PCR machine: DNA template samples with an unknown initial number of copies, and the response of fluorescence incremental curves for DNA quantifications. The control factors were defined based on quantification experiments. The simulations in this study also considered noise factors, including DNA amplification efficiency variations and the chemical reaction instability.
- Coefficient of variation (CV): The effectiveness of the model was verified using experimental data obtained from commercial real-time PCR machines. This study uses inter-assay CV instead of the quadratic loss function to quantify the performance deviation. The coefficient of variation, CV, with the percentage unit is usually employed as an index for DNA quantification. It is defined as:
  (1) $$\mathrm{CV}=-\frac{{\text{log}}_{10}\;(\frac{1}{n}\;\sum_{n}\;{\mathrm{ui}}_{M})-{\text{log}}_{10}\;({\mathrm{ui}}_{s})}{{\text{log}}_{10}\;({\mathrm{ui}}_{s})}\times 100\%$$
  where *ui* denotes the initial number of copies of the test samples, the suffix *M* indicates the measured results of the number of DNA fragments in the test sample. The suffix *s* indicates the correct DNA amount known from the standard sample, collecting n-times measurement, and checking with the correct value. The degree of uncertainty of DNA quantification can be evaluated.
- Parameter diagram (P-diagram): The P-diagram was developed to classify all variables and determine the influential factors based on the numerical model.
- S/N ratio calculation for the smaller the better CV: Taguchi's S/N ratio for the smaller the better CV was implemented to quantify the influences of design factors and analyze the chip design to achieve high reliability. The goal of DNS quantification is to make the CV as low as possible. The S/N ratio can be calculated by:
  (2) $$\mathrm{SN}=-10\cdot {\text{log}}_{10}\;(\frac{1}{n}\;\sum \;\mathrm{CV})$$
  This equation indicates that the S/N ratio can be determined by comparing the log of the summation of the CV values with the instrument arrangements.
- Orthogonal arrays: The orthogonal arrays suggested by Taguchi’s method provide a set of minimum experiments for determining the influential factor for the real-time PCR on-a-chip design.

The systematic approach for the robust design of the real-time PCR on-a-chip consists of four steps:

1. *System modeling*: build a system identification model to simulate the machine performance. The feature of real-time PCR techniques is the fluorescence increments with respect to each reaction cycle. This can be written as:

(3) $$\mathrm{FI}(k)=-\sum_{i=1}^{k-1}\;a_{i}\;\mathrm{FI}(k-i)+\sum_{i=0}^{k-1}\;b_{i}u(k-i)+\xi (k)$$

where *FI(k)* is the fluorescence intensity of the *k* th cycle, which is influenced by the previous cycles. These influences can be calculated by weighting coefficients, *a_i_*. The intensity depends on the DNA fragments concentration, *u*, and not only concerns the current cycle, but also can be correlated to the previous cycle by weighting coefficients, *b_i_*. The concentration, *u*, can be estimated by *u*(*k*)=(1+*η*)*^k^*·*u*(0). Here, *u*(0) denotes the initial number of copies and is the amplification efficiency. The fluorescence intensity readings are disturbed by the noise term, ξ. Assuming that the disturbance is white noise, then Equation (3) can be expressed as:

(4) $$\begin{array}{lll} \mathrm{FI}(k) & = & -a_{1}\;\mathrm{FI}(k-1)-a_{2}\;\mathrm{FI}(k-2)-\cdot \cdot \cdot \cdot \cdot -a_{k-1}\;\mathrm{FI}(1)+b_{0}u(k)+b_{1}u(k-1)+b_{2}u(k-2)\\ & & +\cdot \cdot \cdot \cdot \cdot +b_{k-1}u(1)+\xi (k) \end{array}$$

We can define two matrices as:

$$\Phi_{k}^{T}=\left[\begin{array}{llllllll} \mathrm{FI}(k-1) & \mathrm{FI}(k-2) & \cdot \cdot \cdot \cdot \cdot & \mathrm{FI}(1) & u(k) & u(k-1) & \cdot \cdot \cdot \cdot \cdot & u(1) \end{array}\right]$$

and:

$$\theta^{T}=\left[\begin{array}{llllllll} -a_{1} & -a_{2} & \cdots & -a_{k-1} & b_{0} & b_{1} & \cdots & b_{k-1} \end{array}\right]$$

Equation (2) can be rewritten in a matrix form as follows:

(5) $$\mathrm{FI}(k)=\Phi_{k}^{T}\;\theta +\zeta \;(k)$$

We solve Equation (5) to get the matrix *θ*, and determine all of the weighting coefficients. The real-time PCR machine is formulated as an ideal function: the fluorescence intensity increment versus the DNA fragments concentration of each reaction cycle.

2. *Data collection and prediction confirmation*: To solve Equation (5), we must collect experimental data. The deviation between the experimental data and the model prediction can be expressed by:

(6) $$e(k)=\mathrm{FI}(k)-\Phi_{k}^{T}\;\theta$$

Although Equations (5) and (6) appear similar, the parameters, *ζ*(*k*) and *e*(*k*) have very different meanings. One is a time series of random numbers, while the other is the error function, which can be well defined by the experimental data. Through the error minimization of the least square method, $\frac{\partial e}{\partial \theta }=0$, we can calculate the matrix, *θ*. However, the fluorescence readings of the first several cycles are highly disturbed by noises, and they have no physical bearing on data analysis. To avoid these noise disturbances, we use the weighting coefficients to redefine the error function as:

(7) $$J=\sum_{k=1}^{N}\;w(k)\cdot e^{2}\;(k)$$

The weighting coefficients, *w*(*k*), are expressed by the matrix as follows:

(8) $$W=\left[\begin{array}{cccc} w(1) & 0 & \cdots & 0\\ 0 & w(2) & \cdots & 0\\ \cdot & \cdot & \cdots & \cdot \cdot \\ 0 & 0 & \cdots & w(N) \end{array}\right]$$

We calculate the error minimization again with the weighted error function, *J*. The matrix, *θ*, can be calculated by:

(9) $$\theta ={\left(\Phi^{T}W\Phi \right)}^{-1}\;\Phi^{T}\;W\cdot \mathrm{FI}(k)$$

Through minimizing the errors between model prediction and experimental data, the system model can provide accurate results to simulate the real-time PCR machine.

3. *Design factor analysis*: The P-diagram was developed to correlate the design factors, specifications, and the performance improvements of the lab-on-a-chip system. Numerical experiments were carried out to test these design factors with various specifications. Taguchi’s method was proposed to determine the essential factor. This method provides the systematic experiment arrangement required for the robust design of a real-time PCR-on-a-chip. The critical design factor is identified through the calculation of the S/N ratio defined in Equation (2) for the smaller better CV. The factor with the highest S/N ratio change indicates that it is the most critical since the CV, the coefficient of variation of DNA quantification, is improved the most.

4. *System implementation and performance confirmation*: Referring to the suggestions on design analysis, the prototype of the real-time PCR-on-a-chip can be constructed to test the robustness of the instrument. DNA quantification experiments were conducted for the samples with different initial copies numbered from 10^8^ to 10^4^ copies/mL. The reproducibility of the DNA quantification is tested by inter-assay CV for different initial numbers of copies to verify the performance of the chip prototype. Following the above steps, the proposed methodology provides a systematic approach to achieving the robust design of real-time PCR-on-a-chip.

## 3. Experimental Apparatus

### 3.1. The Real-Time PCR-on-a-chip System

The real-time PCR-on-a-chip system consists of a reactor chip, miniature thermal cycler, and the fluorescence detection system. The reactor chip has a disposable design. Two chip geometries are used in this study.

**Figure 1.**
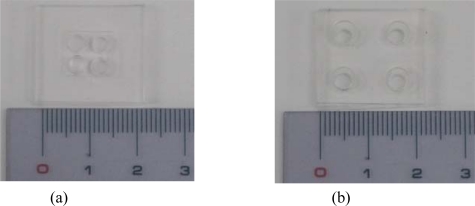

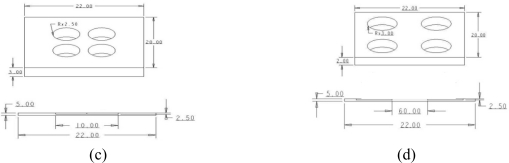
A photograph of the four-well reactor chips with different geometry is shown in (a) and (b). The compact-geometric chip shown in (a) is good for temperature uniformity. As shown in (b), the large-well type is used for high-speed thermal cycling. The schematic drawings of the two types are shown in (c) and (d). Unit: mm

Figures 1(a) and 1(b) show the reactor chips with two different geometries. The test sample volume for DNA quantification is 10 μL. Therefore one cell volume is set at 20 μL. The cells can be either large with a thin hole or thick with a small diameter. The compact geometric chip at the left side of the photograph can provide the four-well DNA samples with good temperature uniformity. At the right side, the large-well type has the large and thin cells. This can yield high heat flux along the vertical direction of the chip and it is good for high-speed thermal cycling. Although the diameter and depth of the sample wells can be arbitrarily determined, they must need the volume requirement and the spacing of the wells should be carefully considered. PCR mixtures must be dispensed to fill the well. For the large diameter well type, the wide spacing is necessary for pipetting operations, especially allowing the transverse motion of the pipette tip while also most importantly avoiding contamination; that is why the large-well type chip has much wider spacing than does the compact one. Figures 1 (c) and (d) show the schematic drawings of these two chips.

**Figure 2.**
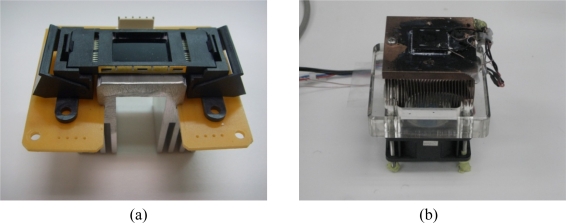
The photograph of the micro heater chip is shown in (a). The fast thermal cycling speed can be achieved. The TEC is shown in (b). The TEC has the large thermal mass and is good for uniform temperature distribution.

The core of the thermal cycler can be selected either as a micro heater chip or a thermoelectric cooler (TEC). The advantage of the micro heater chip is a high thermal cycling speed. TEC employs the Peltier effect, acting as a solid-state heat pump or cooling device for PCR thermal cycling control. The temperature uniformity on the TEC surface can reach to 0.01 °C; however, the heating and cooling rates are both limited within 2 °C/sec. Either a micro heater chip or a thermoelectric cooler (TEC) were considered as the thermal cycling control core for the real-time PCR-on-a-chip system. The micro heater chip used thin-film platinum resistors as the heater. The advantage is the high thermal cycling speed. The high heating and cooling rates of the system reach 20 and 10 °C/sec, respectively. TEC employs the Peltier effect, acting as a solid-state heat pump or cooling device for PCR thermal cycling control. The main benefit of using TEC to heat as well as cool is to enhance the temperature uniformity. The TEC consists of PN junctions, and ceramic plates provide a big thermal mass. The temperature uniformity on the ceramic surface can reach 0.01°C. However, due to the big thermal mass, the heating and cooling rates are both slow and only 1.5 °C/sec can be achieved. Figures 2(a) and (b) show photographs of the micro heater chip and TEC.

Four detectors, including the photodiode; photomultiplier tube (PMT), array or line-charge coupled device (CCD), and cooled CCD, are considered for constructing the fluorescence detection system. LED was widely employed in commercial real-time PCR machines. The light intensity has a 5% variation. White light lamp is also considered as the light source due to its ability to provide multi-wavelength excitation. Compared with LED, the light intensity of the lamp can be controlled within a 3% variation.

**Figure 3.**
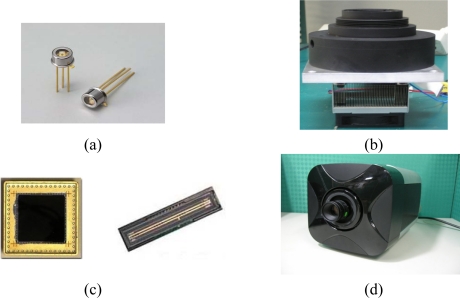
Four detectors were considered in this study for constructing the fluorescence detection system of the real-time PCR on chip. (a) Photodiode component. (b) Assembled PMT module with high-voltage power supply. (c) Line and array CCD chip. (d) CCD camera with cooling devices for low noise detection.

The photodiode detector type has its sensitivity counted by the output current signal per incident light intensity, Amp/Watt. The photodiode used in this study has a 0.2 Amp/Watt sensitivity. The signal to noise (S/N) ratio for fluorescence detection is 32 dB. The dark noise, the mean-square fluctuations to the fluorescence readings, can be counted by electron voltage, eV. The photodiode used in this study has the dark noise of 65 eV. Figure 3 (a) shows a photograph of the photodiode component.

The PMT consists of a vacuum tube. The sensitivity can be high to quantum yield level. The PMT used in this study has a quantum efficiency of up to 30%. The S/N for fluorescence detection is 60 dB, and the dark noise can be as low as 1–3 eV. A photograph of PMT used for this study is shown in Figure 3 (b). The PMT is packaged into a module as shown on the figures. Inside the black casing is the tube. The bottom aluminum plate and heat sink were designed to assemble the high-voltage power supply for PMT.

Line CCD and array CCD are both considered as the detector. This study obtained the chip for the medical applications, and 10^−4^ lux light intensity can be sensed. The S/N for fluorescence detection is 50 dB. The dark noise is 25 eV. Figure 3(c) shows the line CCD and array CCD.

This study also integrated the array CCD with the signal processing circuits and the cooling devices as a CCD camera for fluorescence detection. The cooling devices can keep the CCD chip at a low temperature, down to −15 °C. The S/N can be raised 3 dB to 53 dB, and the dark noise can be reduced to 3∼5 eV. Figure 3 (d) shows this photograph.

**Figure 4.**
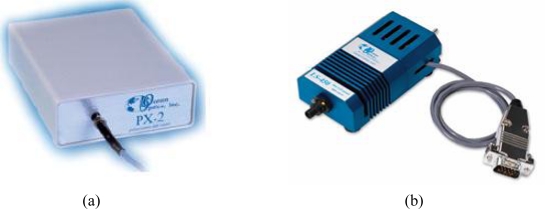
Two excitation light sources were considered for the real-time PCR on-chip system: (a) shows the white light lamp with light-intensity control ability to 3% variation, and (b) shows the LED providing a simple excitation light source with high intensity variation to 5%.

In addition to the fluorescence detector, two excitation light sources, LED or white light lamp, were considered for an on-chip system. LED is widely employed for commercial real-time PCR machines. The light intensity has 5% variation. In this study, we used a 470 nm wavelength for fluorescence excitation. A white light lamp was also considered as the light source due to its ability to provide multi-wavelength excitation, and we can change the excitation light wavelength by sliding filters [14]. Compared with LED, the light intensity of the lamp can be controlled within a 3% variation. Figure 4(a) and (b) shows photographs of these two excitation light sources.

In summary, the variable geometric reactor chip, the miniature thermal cycler with the selectable temperature control core (micro heater chip or TEC), and the fluorescence detection system with detector selections, include a photodiode, PMT, CCD, or cooled CCD. The different excitation light source selections include LED or a white light lamp, which makes up a flexible real-time PCR on-a-chip system. Robust design involves determining the essential factors and selecting suitable design specifications. The real-time PCR-on-a-chip constructed in this study provided flexibility in selecting different system arrangements to meet the requirements of robust design.

### 3.2. DNA Quantification Experiments

This study employed two instruments: the proposed real-time PCR-on-a-chip prototype constructed by us and another commercial product (LightCycler, Roche, USA). These instruments were tested for quantitative measurements on the concentration of HBV SC 11, DNA fragments. The initial number of copies of the test samples ranged from 10^4^ to 10^8^ copies/mL. Serial dilutions of the plasmid ranging from 10^3^ to 10^9^ copies/mL were used to generate the standard curve. LightCycler-Fast Start DNA master with Cat No. 3003230 and SYBR Green I labeling dye were employed as the PCR mixture in all of the runs. The PCR reaction was performed in a total volume of 10 μL, containing 2 μL of DNA template, 1 μL of LightCycler FastStart DNA Master Hybridization Mixture (Taq DNA polymerase, PCR reaction buffer, 10 mM MgCl_2_, and dNTP mixture, Roche Diagnostics Applied Science), 0.8 μL of 25 mM MgCl_2_, 0.3 μM each of the probes, and the 5 μM of each primer. The thermal cycling protocol was as follows: initial hot start denaturation at 95 °C for 10 min, which was followed by 55 cycles of denaturation at 95 °C for 5 sec, annealing at 53 °C for 10 sec, and extension at 72 °C for 20 sec.

### 3.3. Accelerated Life Test

Accelerated life testing uses stress loading to quantify the life characteristics of the system or component. The Arrhenis model [15] is usually used to determine the life test time and the stress loading conditions. This model can be expressed as:

(10) $$\mathrm{AF}=\text{exp}\left\{\frac{\mathrm{Ea}}{B}*\left[\frac{1}{\mathrm{Tu}}-\frac{1}{\mathrm{Ts}}\right]+({\mathrm{RHu}}^{2}-{\mathrm{RHs}}^{2})\right\}$$

where *AF* is the acceleration factor, which is the ratio of the demanded lift time and acceleration test time. Ea is the activity energy with the unit in eV, and *B* is the Boltzman constant. *Tu* and *RHu* are the normal operating temperature 25 °C and the relative humidity 70%, respectively. The terms denoted by the footnote *s* indicate the temperature and humidity set for the acceleration test.

Accelerated life testing uses an environmental test chamber to create a severe environment, hot and humid, to evaluate the life characteristics of a real-time PCR-on-a-chip system. The accelerated life testing chamber can achieve high temperatures from 40 to 200 °C, and the consistency is +/−0.2 °C. Relative humidity control ranges from 60% to 100%, and the consistency is +/−5%.

## 4. Results and Discussion

**Figure 5.**
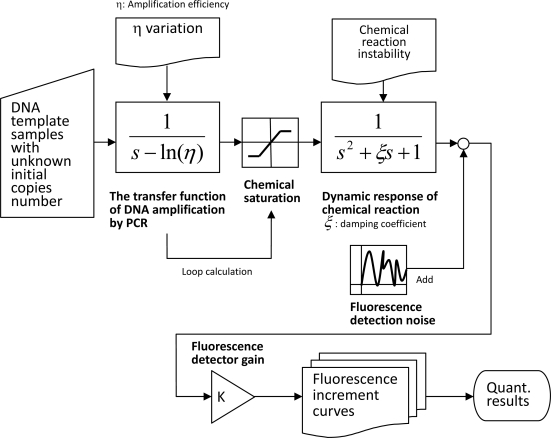
The system identification model developed to simulate the real-time PCR machine and the noise factors are indicated as the DNA amplification efficiency (η) variation, the chemical reaction instability caused different damping coefficient (ξ) and the fluorescence detection noise. With this model, numerical experiments can be conducted to evaluate the instrument design.

The authors have worked with the Roche machine for years. These experimental data were used to build the system model. Equation (9) was solved to derive the matrix by minimizing the prediction errors, and all weighting coefficients of equation (3) could be determined. To simplify the expression of the simulation model, we used the Simulink tools of MATLAB [16] to reformat the polynomial solutions into a system block diagram as Figure 5 shows.

The system block diagram shows that the real-time PCR machine model is primarily divided into two parts: the exponential amplification of DNA fragments and the dynamic response of chemical reaction. The exponential growth is the feature of PCR; the transfer function, $\frac{1}{s-\text{ln}(\eta )}$, can be used to illustrate the types of reactions, where *η* denotes the amplification efficiency. Since it is impossible to infinitely amplify DNA fragments, the model includes a chemical saturation term to set the upper-limit concentration. The fluorescence readings correspond to DNA amplification, displaying the damping effects that may be caused by labeling dye bindings, quantum yields, and chemical instability. A second order system of the transfer function, $\frac{1}{s^{2}+\zeta s+1}$, was coupled with the amplification block diagram to simulate the dynamic response of the chemical reaction, where *ζ* denotes the damping coefficient. The signal is then picked up by the florescence detection system with a gain, K. The fluorescence readings are randomly disturbed by the detection noise. Three noise factors can be identified in the block diagram in Figure 5.

**Figure 6.**
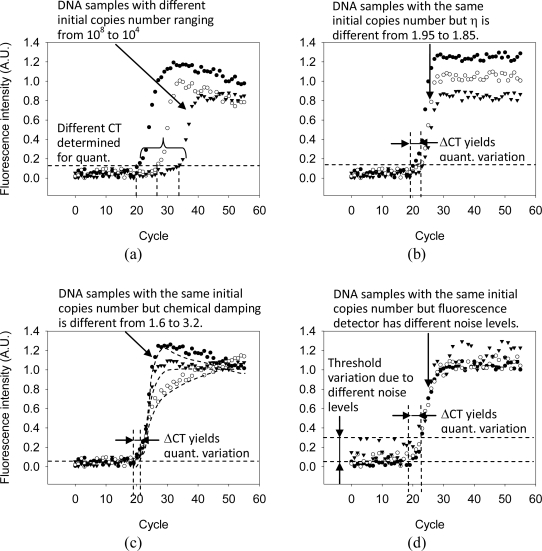
Simulation results of the real-time PCR machine: With respect to the samples of different initial copies number, the fluorescence intensity versus PCR cycle are simulated in (a). The fluorescence readings of each PCR cycle are randomly disturbed by the noise factors, including different amplification efficiency, chemical instability, and fluorescence detection noise level, and their effects are simulated in (b), (c) and (d). All three factors have an impact on the reproducibility of DNA quantification (extracted by quant.) by the variation of the threshold cycle (CT).

The block diagram in Figure 6 shows that the system identification model [13] gave the simulation results of DNA quantification. The fluorescence intensity of each PCR cycle can be predicted with respect to the samples that contain different initial copies. The threshold cycle (CT) is determined as the fluorescence intensity exceeds the background level by 3 dB, and the CT value can be used to determine the initial DNA fragments concentration for quantity analysis. Figure 6(b) shows the different amplification efficiency, *η* yielding the variable fluorescence curves even when the test samples have the same initial number of copies. Figure 6(c) illustrates the chemical instability effects. Figure 6(d) shows the fluorescence detections are randomly disturbed by the noises. Noise intensity levels generate the different fluorescence backgrounds. All these noise factors change the threshold level and make large ΔCT of the test samples with the same initial number of copies.

**Figure 7.**
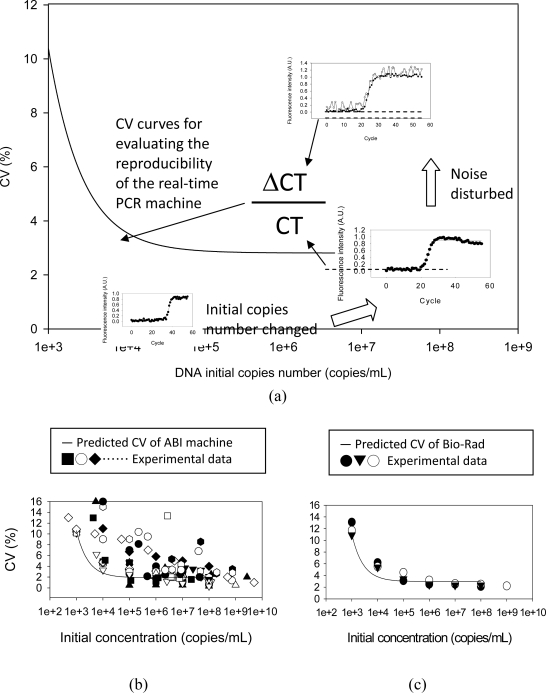

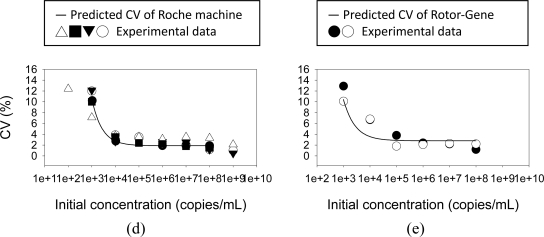
How to draw the coefficient of variation (CV) curves using the simulation results is illustrated in (a). The predicted CV of the ABI, Bio-Rad, Roche, and Rotor-Gene machines and their comparisons to the experimental data are shown in (b), (c), (d), (e).

The coefficient of variation (CV) can be calculated by Equation (1) using CT and ΔCT. Figure 7(a) schematically shows how the CV curve of the real-time PCR machine is drawn using the numerical simulation results. CV is an index for evaluating the reliability of a real-time PCR machine. By adjusting the parameters, the simulation model can be used to predict the CV curves for four commercial machines: ABI 7000 series [17–35], Roche LightCycler [36–42], BioRad iCycler [43–45], and Rotor-Gene [46,47]. It was too difficult to obtain experimental data using four instruments; instead, published data was collected from thirty-four published articles using these four instruments. Figures 7 (b)–(d) shows the prediction results and comparisons to experimental data. These results confirm the numerical prediction and experimental data.

Commercial real-time PCR machines show diversity in the designs of the thermal cycler, the fluorescence detection system, and the related mechanisms [48]. Table 1 lists the specifications and related mechanical design of four commercial instruments from ABI, Bio-Rad, Roche, and Rotor-Gene.

To begin design analysis, five influential factors were selected: the maximum heating/cooling rate, temperature control uniformity, fluorescence detector S/N ratio, mechanical design for fluorescence detection, and the stability of the excitation light source. Table 2 lists the design factors of the real-time PCR machine. The second row in this figure illustrates the influence of each factor on DNA quantification noise. Referring to the commercial machines, the design specifications for thermal cycling speed, temperature uniformity, fluorescence noise detection, mechanical structure, and excitation light sources can be determined.

**Figure 8.**
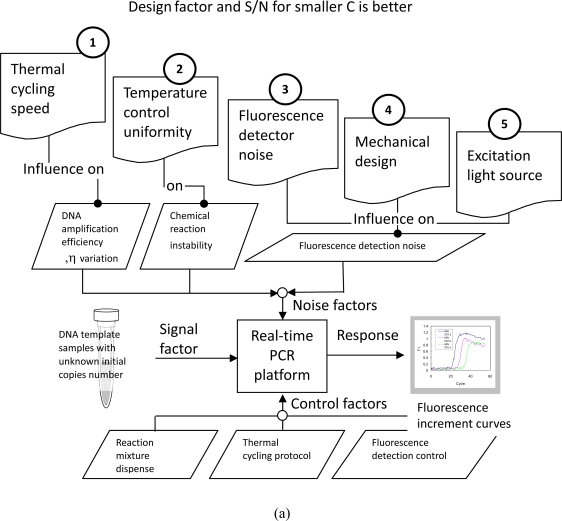

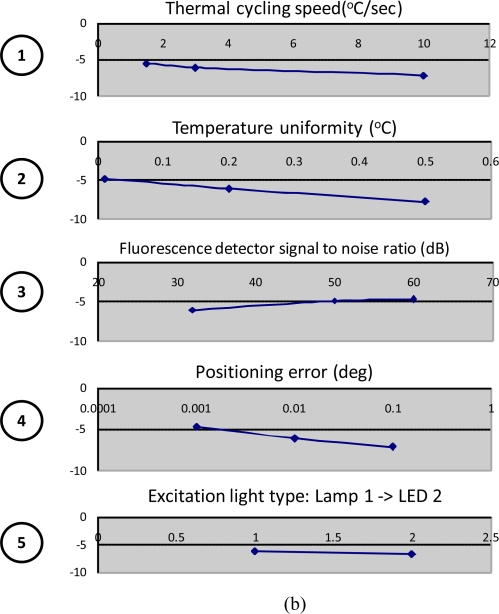
P-diagram of the real-time PCR platform is shown in (a). The design factor analysis results are shown in (b). Based on Taguchi’s method, the design specifications of a real-time PCR on chip were selected by identifying the S/N ratios for smaller CV is better, i.e. a lower coefficient of variation for higher reproducibility of DNA quantification.

Correlating the above factors with the signal, control, and response factors, the P-diagram was developed as shown in Figure 8(a). Five design factors influence the three noise terms. The specifications of each design factor yield different noise levels and impact the reproducibility of DNA quantification. To achieve a robust design, the most influential factor must be identified and the optimized design specification must be selected.

Figure 8(b) shows the results of design analysis. Comparing the amplitudes of S/N ratios, the mechanical design is the most essential factor in the robustness of real-time PCR on-a-chip. Using a no-moving-parts design to eliminate positioning errors is the most effective way to lower CV. Temperature uniformity is the other important factor because it has a higher S/N variation than the other three factors.

**Figure 9.**
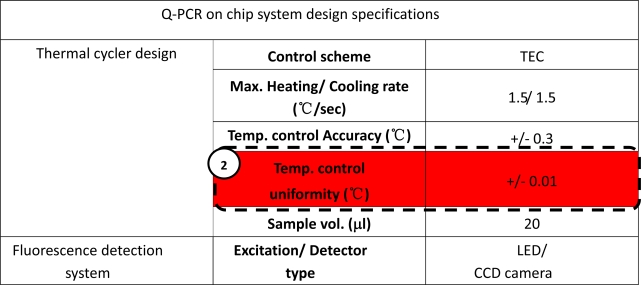

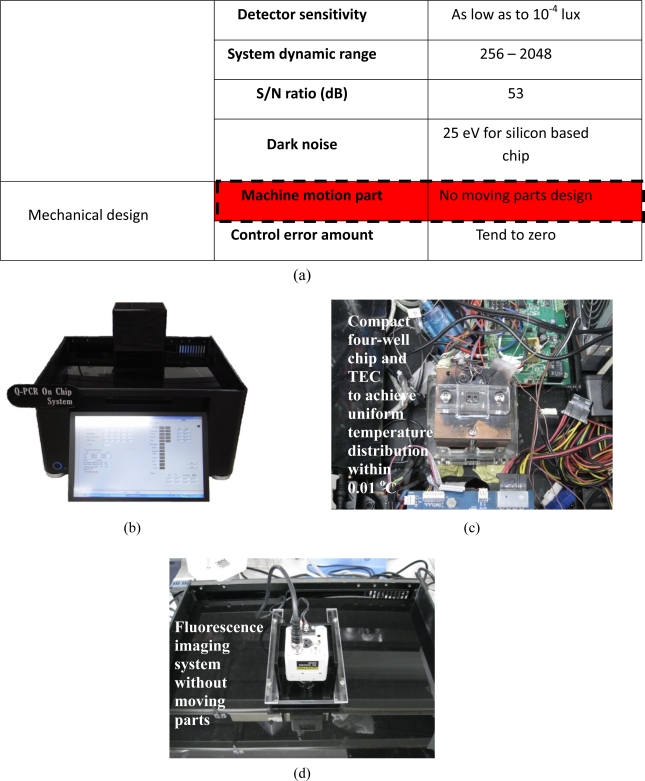
The analyzed results indicate two design parameters are critical: ① no moving part design and ② temperature control uniformity. The design parameters are listed in (a). The real-time PCR on chip prototype is shown in (b). The compact reactor chip design for high temperature uniformity and the fluorescence detection system without moving parts are shown in (c) and (d).

A chip prototype was constructed and tested based on this design analysis. Figure 9(a) lists the design specifications of the lab-on-a-chip system. The specifications of the two major influential factors are no moving-parts design and high-temperature uniformity to +/− 0.01 °C. Figure 9(b) shows a photograph of the on-chip system. TEC and the fluorescence imaging system were employed by the prototype. Referring to Figure 9(c), TEC thermal cycling control with the compact four-well reactor chip produces a uniform temperature distribution. The fluorescence imaging system shown in Figure 9(d) uses a CCD camera to capture fluorescence images of four test samples on the reactor chip.

**Figure 10.**
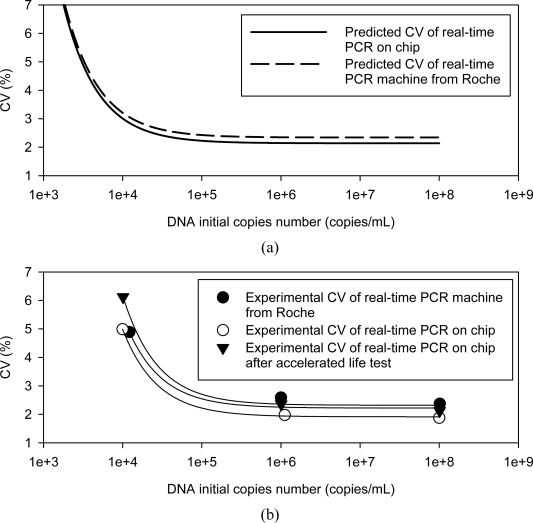
The estimated CV curves of the real-time PCR machine and the on-chip system are shown in (a). The experimental CV curve of the on-chip system is compared with the real-time PCR machine from Roche shown in (b). Even after 3-year operation, the DNA quantification reproducibility of the real-time PCR on chip system still can be compared with that of a commercial real-time PCR machine.

Plasmid HBV samples were used to perform DNA quantification on-a-chip, and these quantification results were then compared with those obtained from the commercial real-time PCR machine. Figure 10 shows the results. At first, the CV curve of the on-chip system was predictable compared with the Roche curve. Figure 10(a) shows that the average CV can be reduced 0.47% compared to the test samples of the initial copies, with numbers ranging from 10^8^ to 10^4^ copies/mL. Then, five inter-assay experiments were carried out on the chip system and the Roche machine. Five unknown DNA samples were analyzed on the lab-on-a-chip system and the CV curves were calculated. Figure 10(b) shows the CV curves of the real-time PCR machine and the lab-on-a-chip system. With respect to the test samples of initial concentrations ranging from 10^8^ to 10^6^ copies/mL, the CV values of the lab-on-a-chip system are lower than the commercial one by 0.52%. This result correlates well with the model prediction.

The life characteristics of the real-time PCR on the chip system were also investigated. Accelerated life testing was used to evaluate the prototype. Referring to Equation (10), the value of the accelerated factor, *AF,* is 9.52. This indicates that three-year-life characteristics of the on-chip system can be obtained through 115 hours of operation in a test chamber at 50 °C and 85% relative humidity.

Figure 10(b) also shows the CV curves of the lab-on-a-chip system after three-years of operation. The CV values are lower than those of the commercial machine, showing that more reliable DNA quantification results can be obtained by the chip prototype even after a long period of operation.

## 5. Conclusions

This study develops a system identification model to simulate the real-time PCR machine, and confirms its performance using experimental data. This model can be successfully used to predict CV values or determine the uncertain distribution of DNA in quantification experiments. Based on the proposed numerical model, the design factors of the machine were analyzed, and the P-diagram was developed to classify all variables and determine the influential factors to increase the reliability of real-time PCR on-a-chip. A chip prototype was then constructed based on the suggestions of the robustness strategy. Accelerated life testing was used to evaluate the life characteristics of the lab-on-a-chip system. The Arrhenis model was also used to determine suitable environment settings and calculate the accelerated factor. One hundred fifteen operation hours in a 50 °C and 85% relative humidity environment can be used to simulate three-year operation in a standard environment. Test results showed that the proposed real-time on-a-chip system achieves lower quantification uncertainty than a real-time PCR machine in a laboratory even after three years of operation. This result not only proves that it is a reliable instrument, but also verifies the effectiveness of the systematic approach to robust design. Instead of improving sensitivity or miniaturizing the device, the goal of this study is to enhance the robustness of the chip-based system. This systematic approach to robust design can be adapted to other biomedical micro-devices to increase the instrument reliability for speeding up commercialization.

## Figures and Tables

**Table 1.** The specifications of commercial real-time PCR machines diverse in thermal cycler, fluorescence detection system, and mechanical design. The most influential factors on the noises of DNA quantification are analyzed- and pointed out below.

**System schematic drawing**	**Instrument model**				
	**ABI PRISM 7000/7700/7900**	**Bio-Rad iCycler series**	**Roche Light-Cycler Ver. 1/2**		**Rotor-Gen 3000**
**Thermal cycler**					
**Control scheme**	Heating block	Heating block	Air cycling		Air cycling
**Max. Heating/Cooling rate (°C/sec)**	1.5/ 1.5	3.3/ 2.0	Ver.1 3.7 / 2.3	Ver.2 3.3 / 3.0	2.5/ 2.5
**Temp. control Accuracy (°C)**	±0.25	±0.3	±0.3	±0.3	±0.25
**Temp. control uniformity (°C)**	±0.5	±0.4	±0.2	±0.15	±0.01
**Sample vol. (μL)**	20–100	10–200	20	10–100	10–100
**Fluorescence detection system**					
**Excitation/Detector type**	White light lamp/ CCD camera	LED/ Array CCD	LED/ Photo-diode		LED/ PMT
**Detector sensitivity**	As low as to 10^−4^ lux	As low as to 10^−4^ lux	100 photons		Q.E to 30%
**S/N ratio (dB)**	53	50	32		60
**Dark noise**	25 eV for silicon based chip	25 eV for silicon based chip	65 eV for silicon base device		1–3 eV
**Mechanical design**					
**Machine motion part**	Sliding filters holder	Sliding filters holder	Carousel sample holder		Carousel sample holder
**Control error amount**	0.01 deg back slash	0.01 deg back slash	0.1 deg position error		0.1 deg position error

**Table 2.** Real-time PCR machine design factors list.

	1		2		3		4		5
Design factors	Thermal cycling speed (Max heating/ cooling rate)		Temperature control uniformity		Fluorescence detector noises		Optical/ mechanic interferences		Excitation light variation
Influence on noise terms	DNA amplification efficiency, η		Chemical reaction instability, *ζ*		Fluorescence detection noise				
Design spec.	20/10 °C/sec (Micro heater chip) 1.5/ 1.5 °C/sec (ABI spec) 3.3/ 2.0 °C /sec (Bio-Rad) 3.3/ 3.0 °C /sec (Roche) 2.5/ 2.5 °C /sec (Rotor-Gene)		0.01 °C (TEC can achieve this limit and it is also the Rotor-Gene spec) 0.15–0.2 °C (Roche spec) 0.4 °C (Bio-Rad) 0.5 °C (ABI)		32 dB Photodiode (Roche spec) 50 dB CCD (Bio-Rad) 53 dB CCD camera, (ABI) 60 dB PMT (Rotor-Gene)		0.001 No moving part design (on chip system) 0.01 (Sliding filters, ABI, Bio-Rad) 0.1 (Carousel type sample holder, Roche, Rotor-Gene)		3% (Lamp by ABI) 5% (LED by Bio-Rad, Roche and Rotor-Gene)
The induced noise levels	Amplification efficiency variation	Corresponding Design Spec	Damping coefficient change	Spec	Noise level	Spec	Noise level	Spec	Noise level
	∼ 0.98 ± 0.1	20/ 10 °C /sec	*ζ* ∼ 2 ± 0.01	0.01 °C	Base line	32 dB detector	X 0.01	No moving parts	+0% LED
	∼ 0.97 ± 0.2	3.3/ 3.0 °C /sec	*ζ* ∼ 2 ± 0.2	0.2 °C	−5 dB	50 dB detector	X 0.1	Sliding filter	−2% Lamp
	∼ 0.96 ± 0.3	1.5/ 1.5 °C /sec	*ζ* ∼ 2 ± 0.5	0.5 °C	−13 dB	60 dB detector	X 1	Carousel sample holder	
